# In-hospital mortality following treatment with red blood cell transfusion or inotropic therapy during early goal-directed therapy for septic shock: a retrospective propensity-adjusted analysis

**DOI:** 10.1186/s13054-014-0496-y

**Published:** 2014-09-12

**Authors:** Dustin G Mark, John W Morehouse, Yun-Yi Hung, Mamata V Kene, Andrew R Elms, Vincent Liu, Dustin W Ballard, David R Vinson

**Affiliations:** Department of Emergency Medicine, Kaiser Permanente, 275 West Macarthur Boulevard, Oakland, CA 94611 USA; Division of Research, Kaiser Permanente Northern California, 2000 Broadway, Oakland, CA 94612 USA; Department of Emergency Medicine, Kaiser Permanente, 27303 Sleepy Hollow Avenue, Hayward, CA 94545 USA; Department of Emergency Medicine, Kaiser Permanente, 6600 Bruceville Road, South Sacramento, CA 95823 USA; Department of Emergency Medicine, Kaiser Permanente, 99 Montecillo Road, San Rafael, CA 94903 USA; Department of Emergency Medicine, Kaiser Permanente, 1600 Eureka Road, Roseville, CA 95661 USA

## Abstract

**Introduction:**

We sought to investigate whether treatment of subnormal (<70%) central venous oxygen saturation (ScvO_2_) with inotropes or red blood cell (RBC) transfusion during early goal-directed therapy (EGDT) for septic shock is independently associated with in-hospital mortality.

**Methods:**

Retrospective analysis of a prospective EGDT patient database drawn from 21 emergency departments with a single standardized EGDT protocol. Patients were included if, during EGDT, they concomitantly achieved a central venous pressure (CVP) of ≥8 mm Hg and a mean arterial pressure (MAP) of ≥65 mm Hg while registering a ScvO_2_ < 70%. Treatment propensity scores for either RBC transfusion or inotrope administration were separately determined from independent patient sub-cohorts. Propensity-adjusted logistic regression analyses were conducted to test for associations between treatments and in-hospital mortality.

**Results:**

Of 2,595 EGDT patients, 572 (22.0%) met study inclusion criteria. The overall in-hospital mortality rate was 20.5%. Inotropes or RBC transfusions were administered for an ScvO_2_ < 70% to 51.9% of patients. Patients were not statistically more likely to achieve an ScvO_2_ of ≥70% if they were treated with RBC transfusion alone (29/59, 49.2%, *P* = 0.19), inotropic therapy alone (104/226, 46.0%, *P* = 0.15) or both RBC and inotropic therapy (7/12, 58.3%, *P* = 0.23) as compared to no therapy (108/275, 39.3%). Following adjustment for treatment propensity score, RBC transfusion was associated with a decreased adjusted odds ratio (aOR) of in-hospital mortality among patients with hemoglobin values less than 10 g/dL (aOR 0.42, 95% CI 0.18 to 0.97, *P* = 0.04) while inotropic therapy was not associated with in-hospital mortality among patients with hemoglobin values of 10 g/dL or greater (aOR 1.16, 95% CI 0.69 to 1.96, *P* = 0.57).

**Conclusions:**

Among patients with septic shock treated with EGDT in the setting of subnormal ScvO_2_ values despite meeting CVP and MAP target goals, treatment with RBC transfusion may be independently associated with decreased in-hospital mortality.

## Introduction

Early goal-directed therapy (EGDT), when applied to emergency department (ED) patients with septic shock, can reduce mortality compared to standardized resuscitation that targets central venous pressure (CVP), mean arterial pressure (MAP) and urine output goals [1]. EGDT can be conceptualized as a central venous oxygen saturation (ScvO_2_)-guided resuscitation protocol, targeting a goal ScvO_2_ of 70% or greater once standardized CVP, MAP and urine output targets have been achieved. ScvO_2_ values below the 70% target can be increased with therapies aimed at decreasing oxygen consumption (VO_2_), such as mechanical ventilation, sedation and/or pharmacologically induced paralysis, and with therapies aimed at increasing oxygen delivery (DO_2_), such as volume expansion, inotropic therapy, red blood cell (RBC) transfusion and vasodilator administration [2-5].

Since the landmark EGDT trial [1], impressive advances have been reported in reducing sepsis-related mortality with implementation of EGDT-based resuscitation bundles [6-10]. However, the incremental benefit of the ScvO_2_-guided DO_2_ augmentation components of these bundles is unclear [9,11-14]. In particular, the ability of RBC transfusions to augment DO_2_ in sepsis appears minimal [14-18], and liberal transfusion strategies in high-risk or critically ill patients may result in increased mortality rates [19-23], though observational propensity-matched data has suggested an inverse association between RBC transfusion and mortality among patients hospitalized with sepsis [15,24]. Physiologic studies of inotropic therapy in septic shock have demonstrated heterogeneous individual responses both in terms of global cardiac performance and microcirculatory perfusion, with some studies even suggesting overall harm [13,25-30].

Given these uncertain and variable associations, we specifically examined a subset of patients with septic shock, treated in the ED, with documented eligibility for DO_2_ augmentation therapies according to an EGDT protocol. Prior research has been limited in this regard since prospective studies of EGDT have reported significantly fewer proportions of patients either eligible for or receiving DO_2_ augmentation therapies as compared to the landmark EGDT trial [10,31-35]. The specific goals of this investigation were to examine associations between EGDT-recommended DO_2_ augmentation therapies (RBC transfusion and inotropic therapy) and in-hospital mortality rates using a propensity-adjusted analysis. We hypothesized that treatment with DO_2_ augmentation therapies would be associated with improved in-hospital survival when adjusting for severity of illness and comorbidities.

## Material and methods

### Study setting and design

We performed a retrospective review of a prospective quality improvement database of ED patients treated for septic shock with EGDT within Kaiser Permanente Northern California (KPNC), an integrated healthcare delivery system, between March 2010 and September 2012. KPNC provides comprehensive care for more than 3.4 million members using an integrated electronic health record (Epic, Verona, WI, USA). Approximately 500 emergency medicine board-certified or board-eligible physicians staff the 21 EDs comprising the KPNC emergency care network. The total annual ED volume for all centers in 2010 was 741,475. Individual ED annual patient census ranged from 17,544 to 65,440 (median 31,726, interquartile range 24,980 to 41,721). All EDs are part of medical centers with adult intensive care units ranging in size from between 8 to 32 beds. Patient-level clinical data is electronically accessible within hierarchical databases as described previously [36].

**Figure 1:**
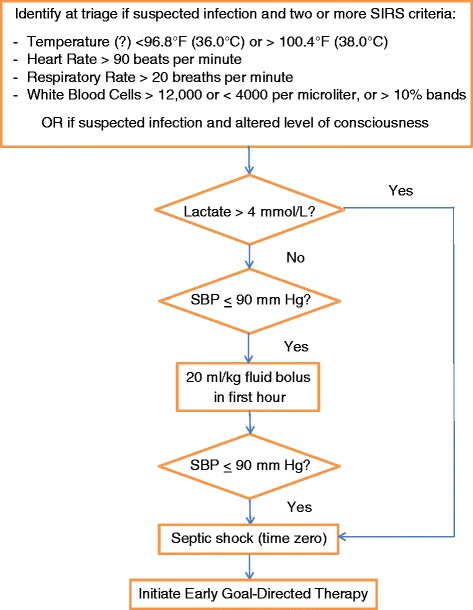
**Early goal-directed therapy eligibility flowchart, Kaiser Permanente Northern California.** Patients presenting with suspected infection and either two or more indicators of a systemic inflammatory response syndrome (SIRS) OR altered mental status are assessed for early goal-directed therapy eligibility based on both (1) initial venous lactate and (2) systolic blood pressure (SBP) as above. WBC, white blood cells.

**Figure 2:**
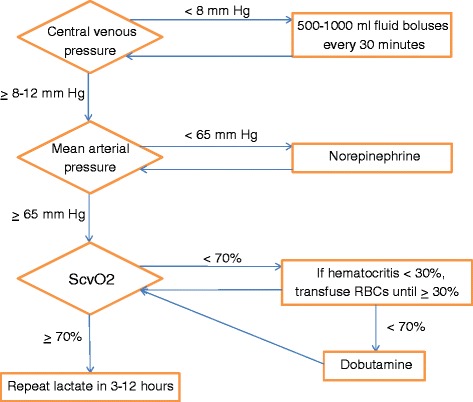
**Early goal-directed therapy treatment flowchart, Kaiser Permanente Northern California.** Patients are considered for central venous oxygenation saturation (ScvO_2_)-guided therapies once their central venous and mean arterial pressures have been optimized with fluid and vasopressor therapies as indicated. Patients with ScvO_2_ values less than 70% are transfused with red blood cells (RBCs) until the hematocrit is 30% or greater, followed by dobutamine administration if ScvO_2_ remains <70%.

The study period followed the uniform implementation of the KPNC EGDT protocol (Figures 1 and 2) as part of a system-wide quality improvement initiative focused on the care of patients with sepsis [7]. Implementation included a training program conducted at each facility emphasizing sepsis diagnoses, management and ultrasound-guided thoracic central venous catheterization. Standardized electronic order sets outlining all elements of the EGDT protocol were in place during the study period. All 21 EDs were capable of using a triple lumen central venous catheter to continuously monitor ScvO_2_ (PreSep Oximetry catheters, Edwards Lifesciences Corporation, Irvine, CA, USA). All RBC products were leukoreduced prior to transfusion. The study was approved by the KPNC Health Services Institutional Review Board (IRB). The IRB waived the requirement for obtaining informed consent from potential study subjects given the minimal risk posed to participants.

### Selection of participants

Electronically recorded data were used to identify patients who simultaneously achieved both a CVP of 8 millimeters mercury (mm Hg) or greater and a MAP of 65 mm Hg or greater (target goals) during the first six hours of eligibility for EGDT. We defined onset of EGDT eligibility in accordance with the ‘time zero’ definition adopted by the KPNC EGDT treatment algorithm as (1) a resulted peripheral blood lactate level of 4 mmol/L or greater or (2) a systolic blood pressure measurement less than or equal to 90 mm Hg following the administration of 20 ml/kg of crystalloid fluids over one hour (Figure 1). Time zero was prospectively recorded by clinical quality abstractors over the course of the study period, with documentation often supplemented by the treating clinical team through the use of an EGDT-specific treatment record.

We included patients if they had two or more ScvO_2_ measurements less than 70% during the first six hours of EGDT eligibility along with adequate arterial oxygenation (SpO_2_ ≥ 93%) and concomitant (within 15 minutes) CVP and MAP values that were at or above target goals. Accepted ScvO_2_ measurements included those obtained by blood drawn from a central venous catheter and PreSep oximetry catheter values that were electronically documented in the patient’s chart. This inclusion strategy was designed to identify patients with relatively clear indications for ScvO_2_-guided therapies as opposed to patients with a single low ScvO_2_ measurement, which may be spurious. Investigators blinded to the study hypothesis manually confirmed inclusion criteria and verified that inotropes (dobutamine, epinephrine or milrinone) and/or RBC transfusions were administered in response to ScvO_2_ measurements below 70% by examining patient-level collated electronic laboratory and hemodynamic data, including medication administration records, supplemented by clinical notes within the electronic health record. Patients under the age of 18 or with pregnancy were excluded from the prospective EGDT database.

### Methods and measurements

Electronically abstracted variables from the first six hours of EGDT eligibility included age, initial heart rate, initial systolic and diastolic blood pressure, initial shock index, total intravenous fluids administered, time to initial antibiotic administration, initial measured ScvO_2_, final measured ScvO_2_ (minimum one-hour interval from the initial ScvO_2_), initial peripheral blood lactate, final peripheral blood lactate (minimum one-hour interval from the initial lactate), percent lactate clearance per hour (the average hourly change between the initial and final lactate measurements, where positive measurements reflect decreasing lactate values), initiation of mechanical ventilation, vasopressor administration (dopamine, norepinephrine, epinephrine, phenylephrine or vasopressin) and the lowest hemoglobin value (nadir). We categorized several continuous variables based on clinically relevant or previously published significant thresholds: final ScvO_2_ < 70% [1], shock index ≥1.0 [37], lactate ≥4.0 mmol/L [1,38], average hourly lactate clearance ≥5% [31] and antibiotic administration within one hour of shock recognition [39]. Hemoglobin values were abstracted as opposed to hematocrit values since clinicians often used a point-of-care test during resuscitation (iStat, Abbott Laboratories, Abbott Park, IL, USA) that reports hemoglobin but not hematocrit values. For the purpose of this study a hemoglobin value of 10 g/dL was considered equivalent to a hematocrit of 30%.

To enable case-mix adjustment we calculated the following morbidity and mortality prediction scores using previously validated approaches: (1) a modified Sequential Organ Failure Assessment (mSOFA) score [40], (2) a summary modified Elixhauser score [41] and (3) a KPNC-specific electronic Simplified Acute Physiology Score III (eSAPS 3) [42].The mSOFA score was calculated using data obtained during the first six hours of EGDT eligibility (six-hour mSOFA score) and during the first 24 hours of hospitalization (24-hour mSOFA score). SOFA scores obtained during initial ED evaluations have previously been validated among patients with sepsis [43]. The summary modified Elixhauser score is a composite score based on International Classification of Diseases, Ninth Revision (ICD-9) codes for 30 categories of comorbidities. The eSAPS 3 score is a validated adaptation of the original SAPS 3 score using electronic data from KPNC patients collected one hour before and one hour following ICU admission. Together and individually the prediction scores demonstrated moderate discrimination when tested against in-hospital mortality rates in this dataset (combined model c statistic = 0.75, individual model c statistics ranged from 0.65 to 0.71).

### Outcomes and analysis

The primary outcome was in-hospital mortality. We examined the degree of association between the dependent variable (in-hospital mortality) and treatments of interest (inotrope administration and RBC transfusion), adjusting for covariates, using multivariate logistic regression [44]. After exclusion of collinearity based on an r^2^ > 0.7, covariates with strong biologic plausibility or a potentially significant association with the dependent variable on bivariate analysis (defined as a *P* value ≤0.1) were included in the initial model, which was then modified in a stepwise fashion. Age and mortality prediction scores were further refined using cubic splines. The final regression model included age, sex, initial lactate, RBC transfusion, inotropic therapy, total intravenous fluids, hemoglobin nadir, vasopressor administration, initiation of mechanical ventilation, percent lactate clearance per hour, shock index, six-hour mSOFA score, Elixhauser score, and final ScvO_2_ ≥ 70%.

In order to further refine this analysis according to a primary indication for RBC transfusion (hemoglobin nadir less than 10 grams per deciliter (g/dL)) or inotrope administration (hemoglobin nadir of 10 g/dL or greater) for treatment of ScvO_2_ values <70% (Figure 2), we divided the cohort based on hemoglobin nadir values during the first six hours of EGDT eligibility. We then determined separate propensity scores for RBC transfusion and inotrope administration using demographic, comorbidity, initial presenting and EGDT treatment period variables in a multivariable logistic regression model. Adjusted *P* values were calculated by regressing the respective propensity score and treatment against each covariate (as the dependent variable). We did not pursue propensity matching given the limited numbers of study subjects. Rather, we performed logistic regression to assess for an association between treatment (with RBC transfusion or inotropic therapy) and the primary outcome within the respective subgroups, adjusting for the propensity score as both a continuous value and as an ordinal variable stratified into propensity quintiles [45]. Baseline or treatment variables with an adjusted *P* value of ≤0.1 were also included in the regression model as potential residual confounders. All final regression models have a minimum of five event outcomes per independent variable with at least thirty total events [46].

Continuous variables are reported as median values with interquartile ranges. Dichotomous variables are reported as percentages. All analyses were performed using Stata v13.1 (StataCorp LP, College Station, TX, USA). Categorical variables were analyzed using chi-square or Fisher’s exact tests. Continuous variables were analyzed using the Student’s *t* test or Wilcoxon rank-sum test. Potential interactions between RBC transfusion, inotropic therapy, lactate clearance per hour (%) and final ScvO_2_ values were analyzed as part of the modeling process. Regression model results are reported as adjusted odds ratios (aOR) with robust 95% confidence intervals (CIs) adjusted for clustering by hospital. Missing variables were imputed by multiple imputation (n = 5) using the mi command in Stata. Multicollinearity was assessed using the variance inflation factor (VIF). An alpha level of 0.05 was used as the threshold for statistical significance.

## Results

There were 2,595 patient encounters in which EGDT was initiated in the ED during the 31-month study period. Of these, 579 met study inclusion criteria, of which seven were repeat encounters (that is readmissions), leaving a total of 572 (22.0%) unique patient encounters eligible for study. A total of 312 ED physicians managed these 572 patients collectively, with mechanical ventilation initiated in 186 (32.5%) patients and vasopressors administered during the treatment period in 335 (58.6%) patients. A median of 4.0 L (interquartile range 3.0 to 5.0 L) of intravenous fluids were given during the six-hour EGDT treatment period, with equal amounts administered to patients with a nadir hemoglobin <10 g/dL versus those with nadirs ≥10 g/dL (4.0 L versus 4.0 L, *P* = 0.09). Ninety-three percent of patients (534/572) had antibiotic therapy initiated within one hour of the septic shock recognition (time zero).

**Figure 3:**
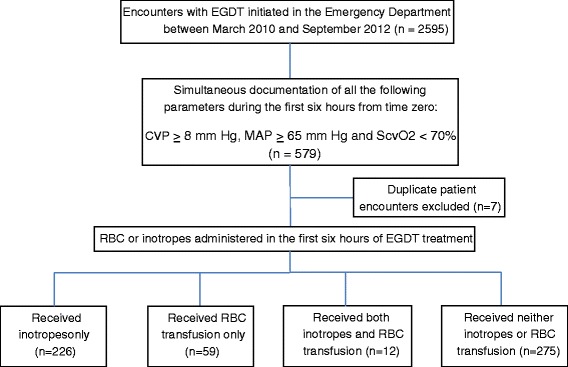
**Study subject selection flowchart.** CVP, central venous pressure; DO_2_, oxygen delivery; EGDT, early goal-directed therapy; MAP, mean arterial pressure; RBC, red blood cell; ScvO_2_, central venous oxygen saturation.

Either inotropic therapy or RBC transfusions were administered to 297 (51.9%) study patients during the six-hour EGDT treatment period. Inotropic therapy was the sole treatment in 226 patients (39.5%), RBC transfusion in 59 patients (10.3%) and both interventions in 12 patients (2.1%) (Figure 3). Among patients receiving inotropic therapy, dobutamine was the sole inotrope in 237 (99.6%) patients (consistent with the KPNC EGDT treatment protocol, Figure 2), with a single additional patient receiving an epinephrine infusion. Median values for age, initial lactate and initial ScvO_2_ were 73 years, 4.4 mmol/L and 63%, respectively. A total of 249/572 (43.5%) patients reached a final ScvO2 of ≥70% by the end of the six-hour EGDT treatment period. Patients were not statistically more likely to achieve an ScvO_2_ of ≥70% if they were treated with RBC transfusion alone (29/59, 49.2%, *P* = 0.19), inotropic therapy alone (104/226, 46.0%, *P* = 0.15) or both RBC and inotropic therapy (7/12, 58.3%, *P* = 0.23) as compared to no therapy (108/275, 39.3%). There was no clinically significant unadjusted difference in the rate of lactate clearance according to treatment provided. It should be noted that the small number of patients receiving both RBC and inotropic therapy limits meaningful analysis of this subgroup. A complete list of abstracted variables grouped by treatment with RBC transfusion or inotropic therapy is presented in Table 1.

**Table 1:** **Patient characteristics and variables according to ScvO**_**2**_ **-guided therapies**

	**Entire cohort (n = 572)**	**Inotrope administered (n = 238)**	**RBCs transfused (n = 71)**	**No intervention (n = 275)**
**Demographics and comorbidities**				
Age, years	73 (62-82)	76 (65-83)	70 (61-81)	71 (60-81)
Female (%)	46.7	47.1	46.5	46.2
Race				
White (%)	57.9	57.1	49.3	59.6
Asian (%)	13.5	15.5	14.1	12.0
Hispanic (%)	12.9	11.8	14.1	13.5
African-American (%)	8.2	7.6	14.1	7.6
Other (%)	7.5	8.0	8.4	7.3
Ischemic heart disease (%)	31.3	30.7	38.0	30.2
Congestive heart failure (%)	36.4	39.5	32.4	34.2
Chronic lung disease (%)	34.1	30.7	26.7	38.9
Diabetes (%)	34.8	34.4	26.7	37.1
HIV/AIDS (%)	0.9	0.4	1.4	1.1
Malignancy (%)	14.9	12.2	32.4	13.1
Rheumatologic disease (%)	6.3	5.0	9.9	6.5
Elixhauser score	16 (8-23)	16 (10-22)	19 (11-28)	15 (7-23)
**Initial presenting variables**				
Systolic blood pressure, mm Hg^*a*^	109 (92-129)	109 (92-129)	102 (86-113)	112 (93-132)
Diastolic blood pressure, mm Hg^*a*^	63 (52-76)	63 (50-76)	56 (44-68)	64 (53-77)
Heart rate, bpm^*a*^	109 (92-125)	108 (91-124)	118 (99-130)	108 (90-124)
Shock index^*a*^	1.0 (0.8-1.2)	1.0 (0.8-1.2)	1.1 (0.9-1.4)	1.0 (0.8-1.2)
Initial serum lactate, mmol/L^*b*^	4.4 (3.0-6.2)	4.5 (3.2-6.3)	4.4 (3.6-7.7)	4.3 (2.8-6.0)
Positive blood cultures (%)	35.3	35.7	49.3	32.4
**EGDT treatment period variables**				
Fluid administered by 6 hours, liters	4.0 (3.0-5.0)	4.0 (3.0-5.1)	4.1 (3.0-5.0)	4.0 (2.6-5.0)
Peak CVP, mm Hg^*b*^	16 (13-21)	16 (13-21)	15 (12-19)	16 (13-22)
Hemoglobin nadir, g/dL^*b*^	11.4 (9.5-13.2)	12.1 (10.3-13.6)	7.9 (7.1-8.8)	11.3 (10.0-13.1)
Antibiotic therapy within one hour (%)	93.4	94.5	95.8	91.6
Mechanical ventilation (%)^*b*^	32.5	32.4	31.0	32.7
Vasopressor administration (%)^*b*^	58.6	72.7	62.0	46.2
Initial ScvO_2_, percent^*b*^	63 (55-69)	62 (55-69)	61 (55-68)	64 (57-69)
Final ScvO_2_, percent^*b*^	68 (61-73)	68 (62-73)	70 (60-75)	67 (61-72)
Final ScvO_2_ > 70% (%)	43.5	46.6	51.3	39.4
Lactate clearance, percent per hour^*b*^	8.4 (4.5-12.9)	8.3 (4.2-12.6)	9.5 (3.6-13.0)	8.3 (4.5-13.0)
Lactate clearance >5% per hour (%)	72.5	72.5	72.4	71.8
mSOFA score at 6 hours	4 (3-6)	5 (4-7)	5 (3-6)	4 (2-6)
eSAPS 3 score	50 (44-57)	52 (46-58)	52 (45-59)	48 (43-55)
**Post-treatment variables**				
mSOFA score at 24 hours	5 (3-7)	5 (4-7)	5 (3-7)	4 (3-6)
In-hospital mortality (%)	20.5	22.7	18.3	19.3

Continuous values are presented as median values with interquartile ranges in parentheses. Dichotomous variables are presented as percentages. ^*a*^At initial emergency department presentation; ^*b*^within the first six hours from shock recognition (time zero). ScvO_2_, central venous oxygen saturation; RBC, red blood cells; HIV/AIDS, human immunodeficiency virus/acquired immunodeficiency syndrome; mm Hg, millimeters mercury; bpm, beats per minute; EGDT, early goal-directed therapy; CVP, central venous pressure; g/dL, grams per deciliter; mSOFA, modified sequential organ failure assessment score; eSAPS, electronic simplified acute physiology score.

There were no significant unadjusted associations between in-hospital mortality and either inotropic therapy (OR 1.33, 95% CI 0.92 to 1.92, *P* = 0.12) or RBC transfusion (OR 0.93, 95% CI 0.52 to 1.66, *P* = 0.80). Multiple logistic regression among the entire cohort adjusted for age, sex, initial lactate, RBC transfusion, inotropic therapy, total intravenous fluids, hemoglobin nadir, vasopressor administration, initiation of mechanical ventilation, percent lactate clearance per hour, shock index, six-hour mSOFA score, Elixhauser score and final ScvO_2_ ≥ 70% likewise did not reveal a statistically significant association between in-hospital mortality and inotropic therapy (aOR 1.10, 95% CI 0.70 to 1.73, *P* = 0.68), but did suggest a trend toward lower mortality among patients transfused with RBCs (aOR 0.58, 95% CI 0.29 to 1.18, *P* = 0.14). Pairwise factorial interactions between RBC transfusion, inotropic therapy, final ScvO_2_ ≥ 70% or lactate clearance ≥5% per hour were not significantly associated with in-hospital mortality when introduced into the regression model, nor did they significantly alter the odds ratio estimates for RBC transfusion or inotropic therapy, suggesting a treatment effect that was independent of reaching these resuscitation endpoints. The VIF was less than four for all independent variables, indicating the absence of multicollinearity,

We then conducted a propensity-adjusted analysis for treatment with RBC transfusion or inotropic therapy among subgroups of patients with a hemoglobin nadir of either less than 10 g/dL or equal or greater than 10 g/dL, respectively. The subgroup characteristics are presented in Tables 2 and 3, stratified by the treatments of interest, along with the covariate *P* values following adjustment for treatment propensity score. The propensity-adjusted regression model estimated that RBC transfusion was significantly associated with decreased in-hospital mortality (aOR 0.42, 95% CI 0.18 to 0.97, *P* = 0.04) among patients with a hemoglobin nadir less than 10 g/dL. However, inotropic therapy was not associated with in-hospital mortality (aOR 1.16, 95% CI 0.69 to 1.96, *P* = 0.57). These results were largely consistent when the model was adjusted using quintiles of propensity score, though the point estimate and CIs were slightly higher resulting in a marginally non-significant result for RBC transfusion (aOR 0.46, 95% CI 0.21 to 1.04, *P* = 0.06, Table 4).

**Table 2:** **Propensity score adjustment among patients eligible for RBC transfusion during EGDT**

	**RBCs transfused (n = 71)**	**No RBCs transfused (n = 108)**	***P*** **value (unadjusted)**	***P*** **value (adjusted)**
**Demographics and comorbidities**				
Age, years	70 (61-81)	72 (60-84)	0.78	0.84
Female (%)	46.5	50.0	0.65	0.83
Race			0.56	0.45
White (%)	49.3	53.7		
Asian (%)	14.1	13.9		
Hispanic (%)	14.1	14.8		
African-American (%)	14.1	7.4		
Other (%)	8.4	10.2		
Ischemic heart disease (%)	38.0	28.7	0.19	0.38
Congestive heart failure (%)	32.4	31.4	0.90	0.67
Chronic lung disease (%)	26.7	25.9	0.90	0.87
Diabetes (%)	26.7	40.7	0.06	0.90
HIV/AIDS (%)	1.4	0.9	0.77	0.71
Malignancy (%)	32.4	15.7	0.01	0.93
Rheumatologic disease (%)	9.9	6.5	0.41	0.51
Elixhauser score	19 (11-28)	16 (9-24)	0.05	0.95
**Initial presenting variables**				
Systolic blood pressure, mm Hg^*a*^	102 (86-113)	107 (92-127)	0.05	0.90
Diastolic blood pressure, mm Hg^*a*^	56 (44-68)	59 (50-70)	0.51	0.54
Heart rate, bpm^*a*^	118 (99-130)	109 (90-123)	0.02	0.21
Shock index^*a*^	1.1 (0.9-1.4)	1.0 (0.8-1.2)	0.001	0.27
Shock index ≥1.0	74.6	51.9	0.003	0.11
Serum lactate, mmol/L^*b*^	4.4 (3.6-7.7)	4.3 (2.5-5.7)	0.10	0.74
Positive blood cultures (%)	49.3	34.2	0.05	0.73
**EGDT treatment period variables**				
Fluid administered by 6 hours, liters	4.1 (3.0-5.0)	4.0 (3.0-5.7)	0.37	0.48
Peak CVP, mm Hg^*b*^	15 (12-19)	16 (13-22)	0.02	0.57
Hemoglobin nadir, g/dL^*b*^	7.9 (7.1-8.8)	9.2 (8.5-9.5)	<0.001	0.47
Inotropes (%)	16.9	37.0	0.005	0.36
Antibiotic therapy within one hour (%)	95.8	93.5	0.52	0.40
Mechanical ventilation (%)^*b*^	31.0	33.3	0.74	0.74
Vasopressor administration (%)^*b*^	62.0	64.8	0.70	0.60
Initial ScvO_2_, percent^*b*^	61 (55-68)	63 (55-69)	0.97	0.87
Final ScvO_2_, percent^*b*^	70 (60-75)	67 (61-71)	0.51	0.36
Final ScvO_2_ > 70% (%)	51.3	39.1	0.12	0.16
Change in ScvO_2_, percent	7 (−2-12)	5 (−4-12)	0.54	0.34
Lactate clearance, percent per hour^*b*^	9.5 (3.6-13.0)	8.5 (3.9-12.0)	0.13	0.09
Lactate clearance ≥5% per hour (%)	72.4	66.5	0.43	0.28
mSOFA score at 6 hours	5 (3-6)	5 (3-7)	0.41	0.94
eSAPS 3 score	52 (45-59)	50 (44-57)	0.35	0.89
**Post-treatment variables**				
mSOFA score at 24 hours	5 (3-7)	5 (4-7)	0.38	0.85
In-hospital mortality (%)	18.3	19.4	0.85	0.12

A subgroup analysis was conducted among patients with a hemoglobin nadir less than 10 g/dL during EGDT. Unadjusted *P* values are reported for the observed variables based on whether RBC transfusion was administered for an ScvO2 less than 70%. Adjusted *P* values are based on bivariate regression analyses comparing the propensity score (for RBC transfusion) and each variable. Continuous values are presented as median values with interquartile ranges in parentheses. Dichotomous variables are presented as percentages.

^*a*^At initial emergency department presentation; ^*b*^within the first six hours from shock recognition (time zero). RBC, red blood cells; EGDT, early goal-directed therapy; HIV/AIDS, human immunodeficiency virus/acquired immunodeficiency syndrome; mm Hg, millimeters mercury; bpm, beats per minute; CVP, central venous pressure; g/dL, grams per deciliter; ScvO_2_, central venous oxygen saturation; mSOFA, modified sequential organ failure assessment score; eSAPS, electronic simplified acute physiology score.

**Table 3:** **Propensity score adjustment among patients eligible for inotropic therapy during EGDT**

	**Inotropes (n = 186)**	**No inotropes (n = 207)**	***P*** **value (unadjusted)**	***P*** **value (adjusted for propensity score)**
**Demographics and comorbidities**				
Age, years	76 (67-84)	72 (61-81)	0.01	0.85
Female (%)	46.8	44.9	0.74	0.58
Race			0.45	0.47
White (%)	58.6	62.3		
Asian (%)	15.1	11.6		
Hispanic (%)	11.8	12.6		
African-American (%)	6.4	8.2		
Other (%)	8.0	5.3		
Ischemic heart disease (%)	31.7	30.0	0.38	0.96
Congestive heart failure (%)	39.8	37.2	0.60	0.91
Chronic lung disease (%)	32.2	42.5	0.04	0.49
Diabetes (%)	34.4	34.8	0.94	0.29
HIV/AIDS (%)	0.5	1.0	0.63	0.66
Malignancy (%)	10.8	12.1	0.68	0.73
Rheumatologic disease (%)	4.8	6.3	0.54	0.57
Elixhauser score	16 (8-21)	15 (7-23)	0.64	0.38
**Initial presenting variables**				
Systolic blood pressure, mm Hg^*a*^	111 (94-130)	113 (95-132)	0.57	0.23
Diastolic blood pressure, mm Hg^*a*^	65 (51-80)	66 (54-78)	0.51	0.31
Heart rate, bpm^*a*^	108 (92-124)	108 (90-125)	0.80	0.50
Shock index^*a*^	1.0 (0.8-1.2)	1.0 (0.8-1.1)	0.85	0.94
Shock index ≥ 1.0	52.7	50.2	0.63	0.74
Serum lactate, mmol/L^*b*^	4.6 (3.4-6.2)	4.4 (3.0-6.2)	0.22	0.47
Positive initial blood cultures (%)	34.4	31.9	0.60	0.40
**EGDT treatment period variables**				
Fluid administered by 6 hours, liters	4.0 (3.0-5.0)	3.5 (2.5-5.0)	0.03	0.34
Peak CVP, mm Hg^*b*^	16 (13-20)	17 (13-22)	0.27	0.45
Hemoglobin nadir, g/dL^*b*^	12.7 (11.6-14.0)	12.2 (10.9-13.7)	0.04	0.11
RBC transfusion (%)	0	0	n/a	n/a
Antibiotic therapy within one hour (%)	95.2	90.8	0.10	0.11
Mechanical ventilation (%)^*b*^	30.6	34.3	0.44	0.58
Vasopressor administration (%)^*b*^	71.5	42.5	<0.001	0.31
Initial ScvO_2_, percent^*b*^	62 (55-70)	64 (57-69)	0.44	0.07
Final ScvO_2_, percent^*b*^	68 (63-72)	68 (61-73)	0.29	0.62
Final ScvO_2_ > 70% (%)	44.4	42.3	0.68	0.80
Change in ScvO_2_, percent	5 (−2-13)	3(−4-10)	0.16	0.06
Lactate clearance, percent per hour^*b*^	8.2 (4.8-13.0)	8.5 (4.8-13.6)	0.40	0.38
Lactate clearance ≥5% per hour (%)	74.1	74.2	0.98	0.89
mSOFA score at 6 hours	5 (4-6)	4 (2-6)	<0.001	0.51
eSAPS 3 score	52 (46-58)	48 (43-55)	0.02	0.71
**Post-treatment variables**				
mSOFA score at 24 hours	5 (4-7)	4 (3-6)	0.001	0.21
In-hospital mortality (%)	23.7	18.8	0.24	0.41

A subgroup analysis was conducted among patients with a hemoglobin nadir of 10 g/dL or greater during EGDT. Unadjusted *P* values are reported for the observed variables based on whether inotropic therapy was administered for an ScvO2 less than 70%. Adjusted *P* values are based on bivariate regression analyses comparing the propensity score (for inotropic therapy) and each variable. Continuous values are presented as median values with interquartile ranges in parentheses. Dichotomous variables are presented as percentages.

^*a*^At initial emergency department presentation; ^*b*^within the first six hours from shock recognition (time zero). ; EGDT, early goal-directed therapy; HIV/AIDS, human immunodeficiency virus/acquired immunodeficiency syndrome; mm Hg, millimeters mercury; bpm, beats per minute; CVP, central venous pressure; g/dL, grams per deciliter; RBC, red blood cells; ScvO_2_, central venous oxygen saturation; mSOFA, modified sequential organ failure assessment score; eSAPS, electronic simplified acute physiology score.

**Table 4:** **Multiple regression and propensity score adjusted odds ratio of in-hospital mortality according to treatment with red blood cell (RBC) transfusion or inotropic therapy during early goal-directed therapy (EGDT)**

**Odds ratio of in-patient mortality**	**RBC transfusion**	***P*** **value**	**Inotrope therapy**	***P*** **value**
Unadjusted (95% CI)	0.93 (0.52-1.66)	0.80	1.33 (0.92-1.92)	0.12
Multivariate regression adjusted (95% CI)	0.58 (0.29-1.18)	0.14	1.10 (0.70-1.73)	0.68
Propensity score (continuous) adjusted (95% CI)	0.42 (0.18-0.97)	0.04	1.16 (0.69-1.96)	0.57
Propensity score (quintile) adjusted (95% CI)	0.46 (0.21-1.04)	0.06	1.21 (0.77-1.91)	0.41

Multivariate logistic regression analysis of the entire cohort was conducted, followed by propensity score adjusted analyses according to the subgroup of hemoglobin nadir during the first six hours of EGDT treatment; patients with a hemoglobin less than 10 g/dL were included in the analysis of RBC transfusion and patients with a hemoglobin of 10 g/dL or greater were included in the inotrope therapy analysis. Standard error and 95% confidence interval (CI) calculations in the regression analyses are adjusted for clustering by hospital.

## Discussion

In this retrospective analysis of a prospectively identified patient cohort with septic shock undergoing EGDT in the emergency department, DO_2_ augmentation therapies (inotropes and/or RBC transfusion) were administered to just over half the patients with EGDT protocol-driven indications for these treatments (ScvO_2_ < 70% after meeting both CVP and MAP target goals). We observed a propensity-adjusted association between RBC transfusion and decreased in-hospital mortality in patients with hemoglobin values less than 10 g/dL, raising the possibility of a beneficial effect of RBC transfusion as recommended by the EGDT treatment protocol. This association was not significantly impacted by the success or failure of reaching a final ScvO_2_ of ≥70%. In contrast, analysis of patients receiving inotropic therapy alone did not reveal a significant association with in-hospital mortality rates. These findings may be interpreted to suggest a mortality benefit from RBC transfusion among a specific subgroup of patients (subnormal ScvO_2_ and hemoglobin less than 10 g/dL) treated early in the course of septic shock, independent of reaching a target ScvO_2_.

Our study is the first we are aware of to revisit the hypothetical independent benefit of RBC transfusion among patients with septic shock treated in the emergency department since the original EGDT trial [1]. RBC transfusion is arguably the most controversial aspect of the EGDT protocol for several reasons. Foremost, there is controversy over the use of RBC transfusions as a means of augmenting tissue oxygen delivery or microvascular perfusion in patients with sepsis [16,18]. Second, there is a fairly consistent and growing body of evidence demonstrating mortality benefits of restrictive transfusion practices in critical and acute illness, though not specifically among patients with septic shock [19-23]. Finally, the exceptionally high rate of transfusion (64%) during the initial six hours of therapy in the original EGDT study [1] has failed to be approximated in any subsequent randomized or observational study of EGDT [10,31-35,47].

However, several recent studies [15,24,48] have challenged the notion that RBC transfusion is harmful in critically ill patients, and have even suggested a mortality benefit among septic patients using propensity-matched analyses, similar to our results [15,24]. Reasons for the observed decreases in mortality following RBC transfusion during septic shock are elusive at best, though may involve the aforementioned contested theory of augmented microvascular oxygen delivery and the interplay between the pro-inflammatory and immunosuppressive effects of blood transfusions [49]. Others have suggested the increasing use of leukoreduced products in the past decade as a possible explanation [48]. Accordingly, physiologic studies of leukoreduced RBC transfusions in patients with sepsis have revealed improvements in microcirculatory flow and oxygen consumption, chiefly among those with subnormal parameters at baseline [18,50]. In addition, the pro-inflammatory effects of leukoreduced blood, as compared to non-leukoreduced blood, appear to be minimal given near undetectable levels of cytokines, suggesting a possible mechanism by which non-leukoreduced products produced harmful effects in older studies [51].

The lack of an observed association between inotropic therapy and in-hospital mortality is consistent with previously published findings from a similar multicenter sepsis patient database analysis [9]. This is despite the fact that the patients treated with inotropic therapy in this cohort received a larger volume of intravenous fluid than those who were not treated with inotropes (median 4.0 versus 3.5 liters, *P* = 0.03). Some studies have even hinted at potential harms resulting from the use of dobutamine in patients with septic shock [13,29]. In part this may be attributable to the highly variable physiologic effects of dobutamine among patients with severe sepsis or septic shock, specifically with regard to indices of microvascular perfusion, even in the presence of hypoperfusion [26-28]. Biochemical studies in septic shock patients have demonstrated positive relationships between dobutamine administration and levels of the pro-inflammatory cytokine tumor necrosis factor (TNF)-alpha that are not explained by illness severity, and which may predispose patients to higher degrees of organ failure [52,53]. Finally, a recent clinical trial of beta-adrenergic receptor blockade during septic shock demonstrated an unexpected large mortality benefit, potentially unmasking the harms of excessive beta-receptor stimulation with inotropic therapy during septic shock [54].

The fact that only 43.5% of the patients in the study cohort successfully reached a final ScvO_2_ of ≥70% highlights the difficulty of translating evidence from rigorous clinical trials into general clinical practice (given that 95% versus 60% of patients met this goal in the experimental and control arms of the original EGDT trial, respectively) [1,55]. Regardless, we did not observe any strong correlations between the specific use of inotropes or RBC transfusions and the likelihood of reaching the ScvO_2_ endpoint, though it is difficult to interpret this finding given the possibility of unmeasured co-interventions and the small number of patients receiving both interventions during the resuscitation period.

### Limitations

As with all retrospective studies, several variables of interest suffer from a lack of standardized data collection. Accordingly, it should be noted that analysis of this data is limited by the possibility of residual confounding or indication bias not accounted for by propensity adjustment. In particular, the lack of consistent intervals for repeated measurements is a notable limitation, most pertinent for the two study inclusion ScvO2 values, the percent lactate clearance and the final ScvO_2_ at the end of the six-hour EGDT treatment period. Since the observed associations between treatment with RBC transfusion or inotropic therapy and in-hospital mortality appear to be largely independent of these measurements this is likely of minor consequence. However, it is possible that the observed associations between treatment and outcomes are more accurately represented by achievement of these resuscitation endpoints than our data suggests.

We also did not control for the use of downstream interventions when adjusting for predictors of in-patient mortality (that is, use of corticosteroids and protective lung ventilation). However, protective lung ventilation is standard practice throughout our medical system with default ventilator orders for pressure-limited, low tidal volume parameters, so significant non-compliance was unlikely. Given that the independent impact of corticosteroid administration on mortality in sepsis is inconclusive [56-59], the omission of this covariate is unlikely to have altered our results significantly.

Likewise we did not fully control for the intensity of treatment with vasopressors or inotropic therapy. The former was partially adjusted for through the use of the cardiovascular component of the mSOFA score. Regarding inotropic therapy, however, it is possible that the failure to observe an association with in-hospital mortality may simply reflect inadequate dosing or titration. This is suggested by the marginal improvement in ScvO_2_ values observed in the inotropic therapy treatment group. Accordingly, it should be noted that this study was not designed to validate or repudiate the physiologic concepts underlying EGDT.

## Conclusions

Among patients with septic shock treated with EGDT in the setting of subnormal ScvO_2_ values despite meeting CVP and MAP target goals, treatment with RBC transfusion may be independently associated with decreased in-hospital mortality. Treatment with inotropic therapy does not appear to be significantly associated with in-hospital mortality, though conclusions are limited by lack of adjustment for intensity of treatment. Randomized studies addressing the utility of specific ScvO_2_-guided therapies would be useful to both address controversial elements of EGDT and further enable septic shock treatment protocols to be refined for optimal efficacy and efficiency.

## Key messages

- DO_2_ augmentation therapies (RBC transfusion or inotropic therapy) were not consistently given to patients with subnormal ScvO_2_ values in this EGDT patient cohort
- RBC transfusion for subnormal ScvO_2_ values (<70%) during EGDT appears to be at least marginally associated with decreased in-hospital mortality, independent of meeting the ScvO_2_ target goal of 70%
- The administration of inotropic therapy for subnormal ScvO_2_ values during EGDT does not appear to be associated with reduced in-hospital mortality
- Randomized studies of RBC transfusion in early septic shock are needed.

## Abbreviations

aOR: adjusted odds ratio
bpm: beats per minute
CI: confidence interval
CVP: central venous pressure
DO_2_: oxygen delivery
ED: emergency department
EGDT: early goal-directed therapy
eSAPS 3: electronic simplified acute physiology score III
g/dL: grams per deciliter
HIV/AIDS: human immunodeficiency virus/acquired immunodeficiency syndrome
IRB: institutional review board
KPNC: Kaiser Permanente Northern California
MAP: mean arterial pressure
mm Hg: millimeters mercury
mSOFA: modified sequential organ failure assessment score
RBC: red blood cell
ScvO_2_: central venous oxygen saturation
SpO_2_: peripheral blood oxygen saturation
TNF: tumor necrosis factor
VIF: variance inflation factor
VO_2_: oxygen consumption
